# Versatility of Intermittent Abdominal Pressure Ventilation in a Case of Complicated Restrictive Respiratory Failure and COVID-19

**DOI:** 10.3390/healthcare10061012

**Published:** 2022-05-31

**Authors:** Francesca Simioli, Anna Annunziata, Antonietta Coppola, Ediva Myriam Borriello, Sara Spinelli, Giuseppe Fiorentino

**Affiliations:** Department of Respiratory Pathophysiology and Rehabilitation Monaldi–A.O. Dei Colli, 80131 Naples, Italy; anna.annunziata@gmail.com (A.A.); antonietta.coppola84@gmail.com (A.C.); myriam.borriello@gmail.com (E.M.B.); spinelli.sara@outlook.it (S.S.); giuseppefiorentino1@gmail.com (G.F.)

**Keywords:** hypercapnia, mucociliary clearance, high flow nasal cannula, non-invasive ventilation, pneumonia, kyphoscoliosis

## Abstract

Background: The intermittent abdominal pressure ventilation (IAPV) is a non-invasive ventilation (NIV) technique that avoids facial interfaces and is a diurnal ventilatory support alternative for neuromuscular patients during stable chronic phases of the disease. Coronavirus disease 2019 (COVID-19) is a novel infection possibly causing acute respiratory distress syndrome (ARDS). Neuromuscular diseases (NMD) and preexisting respiratory failure can be exacerbated by respiratory infection and progress to severe disease and ICU admission with a poor prognosis. Aim: To report on the versatility and feasibility of IAPV in acute restrictive respiratory failure exacerbated by COVID-19. Patient: We describe the case of a 33-year-old man with spastic tetraparesis, kyphoscoliosis, and impaired cough, eventually leading to a restrictive ventilation pattern. COVID-19 exacerbated respiratory failure and seizures. An NIV trial failed because of inadequate interface adhesion and intolerance. During NIV, dyspnea and seizures worsened. He underwent a high flow nasal cannula (HFNC) with a fluctuating benefit on gas exchange. IAPV was initiated and although there was a lack of cooperation and inability to sit; the compliance was good and a progressive improvement of gas exchange, respiratory rate, and dyspnea was observed.Conclusions: IAPV is a versatile type of NIV that can be adopted in complicated restrictive respiratory failure. COVID-19 exacerbates preexisting conditions and is destined to be a disease of frailty. COVID-19 is not a contraindication to IAPV and this kind of ventilation can be employed in selected cases in a specialistic setting. Moreover, this report suggests that IAPV is safe when used in combination with HFNC. This hybrid approach provides the opportunity to benefit from both therapies, and, in this particular case, prevented the intubation with all connected risks.

## 1. Introduction

Non-invasive ventilation (NIV) is widely indicated for ventilatory pump failure. It improves dyspnea, hypoventilation, and quality of life [1,2]. NIV reduces the need for intubation and prolongs the tracheostomy free time in neuromuscular diseases (NMD) [3]. The interface plays a key role in NIV and determines efficacy and compliance in the short and long term. Since neuromuscular patients can become ventilator-dependent over time, the choice of the interface and the rotation of masks is recommended. Despite this, interface inadequacy or intolerance is the main cause of NIV failure [4].

Intermittent abdominal pressure ventilation (IAPV) is an NIV technique that avoids facial interfaces and has been successfully used as diurnal ventilatory support for neuromuscular patients during stable chronic phases of the disease [5]. The intermittent abdominal pressure ventilator (Dima Italia Srl) delivers up to 2.5 L of air with a modifiable pressure into an elastic sac that is incorporated in a belt. The belt is placed around the abdomen and is cyclically inflated by the ventilator ensuring the diaphragm elevation and exhalation of air in the lungs. At the cessation of air delivery, the belt deflates and the diaphragm descends because of gravity, facilitating inhalation. Any inspiratory capacity of the subject adds volumes of air to those taken mechanically, up to 1200 mL [6].

Coronavirus disease 2019 (COVID-19) is a novel infection possibly causing acute respiratory distress syndrome (ARDS) and early intensive care unit (ICU) admission. The disease has a very variable presentation and progression. According to data from Wuhan in China, an estimated 14% of cases experience a severe illness, and 5% of cases progress to a critical disease requiring treatment in the ICU [7]. The case fatality rate decreased in Italy from 14% during the first wave to 2–3% reported in 2022 [8]. This is likely related to the widespread diffusion of vaccination; unfortunately, frail subjects are still at risk. NMD and preexisting respiratory failure can be exacerbated by respiratory infection and progress to severe disease and ICU admission.

## 2. Case Report

We describe the case of a 33-year-old man affected by acute respiratory failure and COVID-19. The patient is affected by spastic tetraparesis and a severe neuro-psychomotor developmental delay consequent to perinatal asphyxia. Our patient shows kyphoscoliosis, spasticity, hypotonia, impaired cough, and retained tracheobronchial secretions, eventually leading to a restrictive ventilation pattern. In addition, epilepsy contributes to the global complexity and limits cooperation. The patient’s weight is 47 kg.

**Figure 1:**
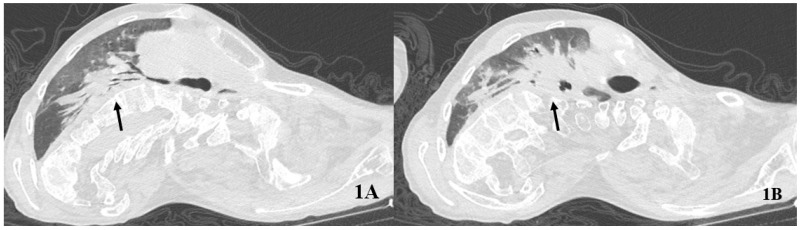
Baseline computed tomography of the chest. The arrows indicate an extensive consolidation from the right hilum (**A**) to the right lower lobe (**B**).

COVID-19 caused fever, respiratory difficulties, and seizures at the onset. The subject was unvaccinated for SARS-CoV-2. He accessed the emergency room and underwent blood gas analysis showing a partially compensated hypercapnic respiratory failure (pH 7.38, pCO_2_ 79 mmHg, pO_2_ 56 mmHg, lactate 0.8 mmol/L, HCO_3_^−^ 38.3 mmol/L). The high-resolution computed tomography (HRCT) of the chest showed ground-glass opacities and consolidations bilaterally (Figure 1). Atelectasis of the right lower lobe was also described.

Blood tests indicated a neutrophilic leukocytosis with elevated inflammatory markers. C-reactive protein was 4 mg/dL, procalcitonin was 0.9 ng/mL. Electrolytic disorders were researched highlighting low potassium and magnesium. The anti-Spike IgG were absent. A pharmacological regimen was initiated with Sotrovimab, systemic steroid, low molecular weight heparin (LMWH), vitamin C, potassium, magnesium, antiepileptics, and supportive therapy. Since the frailty, an extended panel of microbiological surveillance was performed at baseline. Cultures from blood, urine, and intestinal tract were negative. On the third day of hospitalization, Pseudomonas A. and Staphylococcus A. were isolated with a significant bacterial load on tracheobronchial secretions. Intravenous Ceftobiprole was added.

Simultaneously we administered a high-flow nasal cannula (HFNC). The device was set with a temperature of 37 °C, a flow of 45 L/min, and an FiO_2_ of 28%. The patient tolerated well the HFNC up to 12 h daily. The heated and humidified high flow was set to facilitate mucociliary clearance and provide an adequate airflow based on the body size. In addition, we promptly started a physiotherapy and rehabilitation targeted program. This included a daily cough assist device used in between the HFNC sessions. 

After 5 days from hospital admission, we observed a stable improvement of oxygenation but a worsening of pH (7.33) and hypercapnia (97 mmHg). Blood tests, including inflammatory markers, improved. On the contrary, dyspnea and seizures were difficult to manage. At this point, we performed an NIV trial. We set a pressure support ventilation (PSV) mode with the following settings: EPAP 4 cmH_2_O, PS 8 cmH_2_O, rise time 80%, and expiratory trigger 50% (I:E ratio 1:2.4). The patient reached good synchrony with the ventilator, thus reaching a satisfactory tidal volume and respiratory frequency. Unfortunately, it was impossible to guarantee the fit of the interface in the long term. Several types and sizes of masks were tried without success. The blood gas revealed a further worsening, along with seizures, feeding difficulties, and oral secretions.

At this point, we considered the possibility of intubation with a multidisciplinary team. The risk of difficult intubation and periprocedural complication was very high due to a reduced mouth opening and a small range of motion of the neck and mandible, not considering a distorted tracheal anatomy. Overall, the prognosis was poor with invasive mechanical ventilation.

The IAPV trial was performed on the 10th day. The settings were: belt pressure 20 cmH_2_O, respiratory time 1.5 s, frequency 14 pm, and rise time 1. The belt was applied in sessions of 3 to 5 h, twice daily. IAPV consented to enteral nutrition, and it was applied after gastric digestion. At the time the patient was bedridden and the IAPV was well tolerated with bed inclination up to 20°. We did not observe overdistention of the enteric tract or regurgitation. Noteworthy is that the subject received at the time a 4-drug regimen for epilepsy and totally lacked cooperation. During the application of IAPV, he continued the HFNC therapy, thus taking advantage of a hybrid approach.

**Figure 2:**
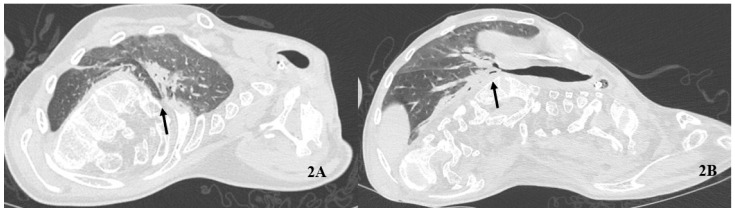
Follow-up computed tomography of the chest. The arrows indicate the sub-total resolution of consolidations at hilum (**A**) and the right lower lobe (**B)**.

A gradual but stable improvement in gas exchange was observed over 14 days. pO_2_ was higher during IAPV plus HFNC compared to HFNC alone. pH and pCO_2_ constantly improved after the IAPV sessions (Table 1). The following HRCT confirmed a substantial resolution of most consolidation bilaterally (Figure 2). 

Overthe days, the status epilepticus improved and a gradual de-escalation of therapy was performed with success. Two of the four drugs were discontinued. The alternation of IAPV + HFNC and HFNC alone also allowed enteral nutrition by a naso-gastric tube with good compliance, which prevented weight loss; the IAPV was performed 2 h after feeding. Finally, SARS-CoV-2 tested negative after 27 days of hospitalization.

No major complications were observed during IAPV. The patient had a pressure injuryin the location of the left concavity of his scoliotic torso; remarkably, the application of IAPV did not worsen the lesion as far as our available follow-up showed.

## 3. Discussion

IAPV is a dated type of non-invasive ventilation (NIV) recently renovated. The technological advancement guarantees reliability and ease of use by healthcare personnel and caregivers. Despite this, IAPV is still considered a niche product. A specialist’s knowledge is required to apply it in clinical practice and to target the patients adequately. In fact, IAPV aims at a specific group of restrictive respiratory failure. Neuromuscular diseases, chest wall abnormalities, kyphoscoliosis, paresis, diaphragm weakness, or respiratory muscle paralysis are suggested indications [9,10], especially when other NIV types are impossible. Nevertheless, the presence of concomitant diseases should not discourage its application. COVID-19 is a novel infectious disease that typically affects the respiratory tract causing pneumonia, respiratory failure, and acute respiratory distress syndrome. There is no evidence of IAPV use in COVID-19. This case focuses on the relevance of IAPV in selected cases of complicated respiratory failure. During IAPV we observed the improvement of gas exchange, a more regular pattern of ventilation, respiratory rate, and less dyspnea. Seizures, enteral nutrition, and anatomical abnormalities were limiting conditions to more conventional ventilation. The patient also showed limits to IAPV such as poor cooperation, impaired motility, and inability to stand or sit; remarkably, it was feasible and well tolerated despite the complexity of the case. Moreover, this report suggests that IAPV is safe when used in combination with HFNC. This hybrid approach provided the opportunity to benefit from both therapies and prevented the intubation with all connected risks in this peculiar case.

## 4. Conclusions

This report suggests that IAPV is a versatile type of NIV that can be adopted in multifactorial respiratory failure. Restrictive patterns and anatomical abnormalities of the chest trachea and face can substantially limit NIV with the most common interfaces. COVID-19 exacerbates preexisting conditions and is destined to be a disease of frailty. COVID-19 is not a contraindication to IAPV and this kind of ventilation can be employed in selected cases in a specialistic setting. Further studies and reports are needed to clarify the role of IAPV in COVID-19.

## Figures and Tables

**Table 1:** Blood gas among days with different ventilation strategies. PSV: pressure support ventilation, HFNC: high flow nasal cannula, IAPV: intermittent abdominal pressure ventilation.

DAY	8	9	10	10	13	13	15	15	18	18
Therapy	PSV	HFNC	HFNC	**IAPV** **+HFNC**	HFNC	**IAPV** **+HFNC**	HFNC	**IAPV** **+HFNC**	HFNC	**IAPV** **+HFNC**
pH	7.35	7.36	7.37	**7.39**	7.37	**7.41**	7.34	**7.45**	7.43	**7.42**
pCO_2_	93	83	79	**77**	76	**70**	85	**66**	73	**65**
pO_2_	86	52	69	**88**	78	**87**	77	**72**	65	**74**
HCO_3_^−^	51.3	46.9	45.7	**46.6**	43.9	**44.4**	45.9	**45.9**	48.5	**42.2**
HCO_3_^−^ std	41.5	38.9	38.3	**39.2**	37.1	**38.0**	38.0	**39.6**	40.9	**36.7**
PF	287	200	216	**275**	260	**290**	233	**257**	232	**264**
FiO_2_	30	26	32	**32**	30	**30**	33	**28**	28	**28**
